# Gut-kidney signaling is impaired in individuals with type 2 diabetes

**DOI:** 10.1093/ckj/sfag315

**Published:** 2026-09-12

**Authors:** Charlotte M Mosterd, Britt E Wever, Michiel J B van Baar, Marcel H A Muskiet, Bert-Jan H van den Born, Max Nieuwdorp, Daan J Touw, Ewout J Hoorn, Liffert Vogt, Daniël H van Raalte

**Affiliations:** Diabetes Center, Department of Internal Medicine, Amsterdam University Medical Center, Amsterdam, The Netherlands; Department of Vascular Medicine, Amsterdam UMC, Amsterdam, The Netherlands; Diabetes Center, Department of Internal Medicine, Amsterdam University Medical Center, Amsterdam, The Netherlands; Department of Vascular Medicine, Amsterdam UMC, Amsterdam, The Netherlands; Diabetes Center, Department of Internal Medicine, Amsterdam University Medical Center, Amsterdam, The Netherlands; Diabetes Center, Department of Internal Medicine, Amsterdam University Medical Center, Amsterdam, The Netherlands; Department of Medicine, Division of Endocrinology, Leiden University Medical Center, Leiden, The Netherlands; Department of Vascular Medicine, Amsterdam UMC, Amsterdam, The Netherlands; Department of Vascular Medicine, Amsterdam UMC, Amsterdam, The Netherlands; Division of Pharmacology, Rijksuniversiteit Groningen, Groningen, The Netherlands; Division of Nephrology and Transplantation, Department of Internal Medicine, Erasmus MC, University Medical Center Rotterdam, Rotterdam, The Netherlands; Department of Nephrology, Amsterdam UMC, Amsterdam, The Netherlands; Diabetes Center, Department of Internal Medicine, Amsterdam University Medical Center, Amsterdam, The Netherlands; Department of Vascular Medicine, Amsterdam UMC, Amsterdam, The Netherlands

## Abstract

**Background and aims:**

In healthy individuals, oral sodium loading induces more rapid natriuresis than intravenous administration, suggesting a gut-kidney feed-forward mechanism. Whether this physiological response is impaired in type 2 diabetes (T2D), and whether pharmacological glucagon-like peptide-1 receptor agonism can restore it, remains unknown.

**Materials and Methods:**

We conducted a mechanistic, randomized, double-blind, placebo-controlled cross-over trial, in men with T2D. Participants adhered to a standardized sodium intake (<90 mmol/day) for 7 days before each study visit. Each participant underwent three experimental conditions in random order: (1) intravenous sodium loading (ISL), (2) oral sodium loading with placebo (OSL-placebo) infusion, and (3) OSL with concomitant exenatide infusion (OSL-exenatide). Each sodium load contained 154 mmol sodium. The primary endpoint was cumulative urinary sodium excretion over 24-h. Secondary endpoints included early natriuresis, fractional sodium excretion, ambulatory blood pressure, extracellular volume, and measured glomerular filtration rate.

**Results:**

Eleven men with T2D (mean age 60 ± 4.7 years, BMI 31.9 ± 3.9 kg/m^2^, HbA1c 7.5 ± 0.9%, systolic blood pressure 127 ± 22 mmHg) completed the study. Median cumulative 24 h sodium excretion did not differ significantly between ISL (105; IQR 55–156 mmol), OSL-placebo (101; 71–134 mmol), or OSL-exenatide (100; 63–123 mmol). Early natriuretic responses and fractional sodium excretion were similar across interventions. Daytime and nighttime blood pressure did not differ between interventions.

**Conclusion:**

In contrast to observations in healthy individuals, OSL did not result in elevated natriuresis compared with ISL in T2D, consistent with impaired gut-kidney feed-forward regulation. Acute GLP-1R-activation with exenatide did not restore this response, suggesting additional entero-renal pathways mediate postprandial natriuretic regulation.

## Graphical Abstract

**Graphical Abstract:**
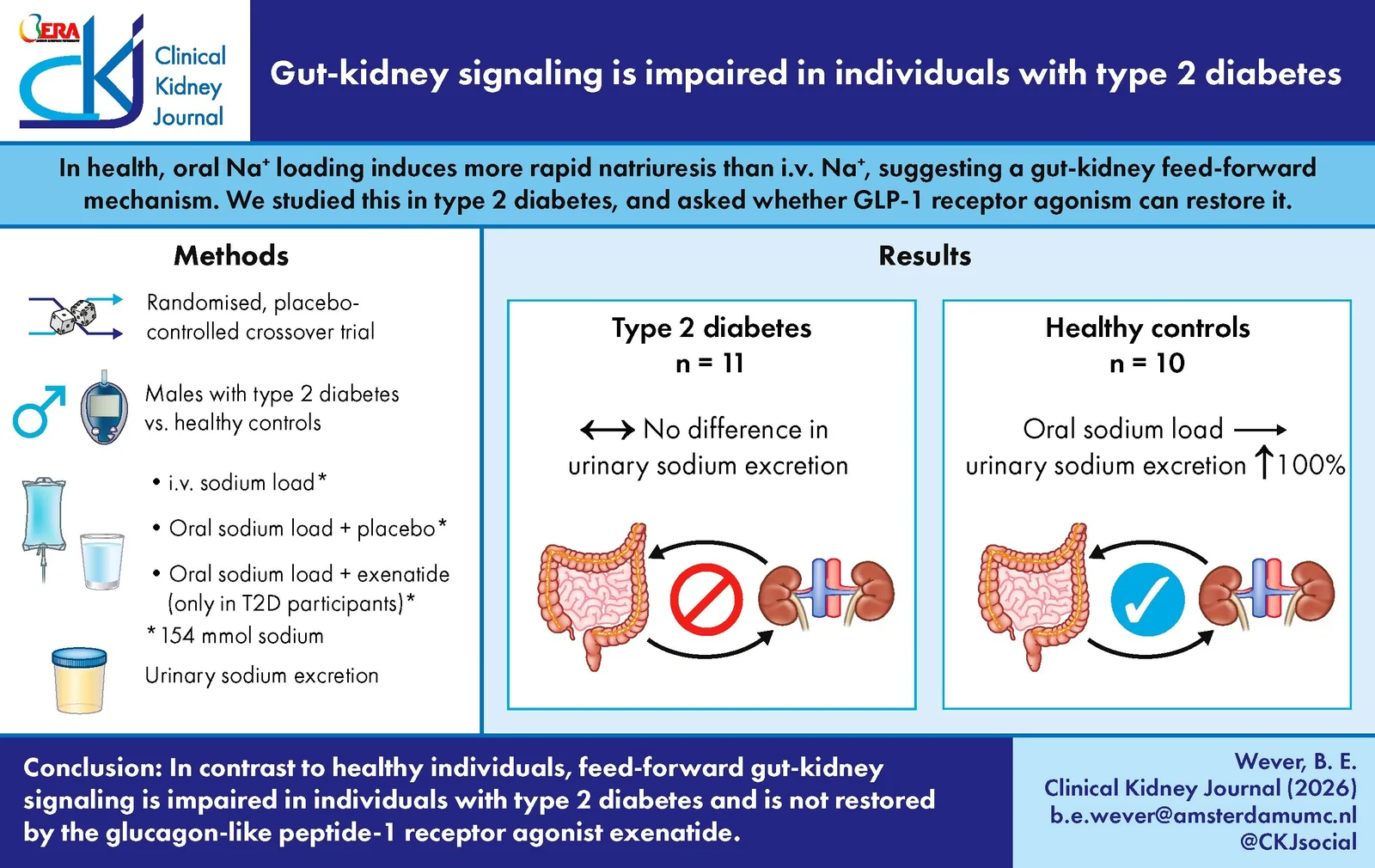

KEY LEARNING POINTS **What was known:**

- In healthy individuals, intact gut-kidney feed-forward signaling enhances renal sodium excretion after oral versus intravenous sodium load.
- In type 2 diabetes, renal sodium handling and blood pressure regulation are often disturbed, but it was unknown whether the gut-kidney feed-forward sodium pathway itself is impaired.
- This feed-forward system is regulated by enteroendocrine hormones, such as GLP-1 hormone. We hypothesized that the feed-forward loop between the gut and the kidney would be impaired in people with type 2 diabetes and that GLP-1 administration could restore this defect.

**This study adds:**

- Our study is the first to demonstrate that feed-forward sodium sensing from the gastrointestinal tract to the kidneys is impaired in individuals with type 2 diabetes.
- In this acute setting, the GLP-1 receptor agonist exenatide did not restore the impaired gut-kidney feed-forward sodium pathway in type 2 diabetes individuals, suggesting that this physiological pathway possibly depends on broader enteroendocrine and neural networks rather than GLP-1 signaling alone.

**Potential impact:**

- An impaired gut-kidney feed-forward sodium pathway in individuals with type 2 diabetes may contribute to inadequate urinary sodium excretion and, in turn, to increased risk of hypertension and cardiovascular disease in susceptible patients.
- Clarifying the mechanisms governing gut-kidney communication could redefine our understanding of sodium regulation in metabolic disease and support the development of targeted therapies designed to restore physiological sodium sensing, with potential benefits for salt-sensitive hypertension and cardiorenal protection.

## INTRODUCTION

Excess dietary sodium intake (>5 g daily) is strongly associated with hypertension and increased cardiokidney risk, including in individuals with type 2 diabetes (T2D) [1]. While the mechanisms linking sodium intake to adverse outcomes remain incompletely understood, salt-sensitive individuals, including those with T2D or chronic kidney disease, exhibit extracellular volume (ECV) expansion and elevated blood pressure in response to sodium excess [2–4]. In addition to volume-mediated effects, high sodium intake may directly cause endothelial dysfunction [5].

The gastrointestinal tract has emerged as an important endocrine organ that may contribute to sodium homeostasis through a so-called gut-kidney axis [6–8]. In sodium-depleted healthy individuals, an equivalent sodium load is excreted more rapidly when administered orally versus intravenously, independent of circulating atrial natriuretic peptide and aldosterone levels [6–8]. This observation suggests the existence of a feed-forward signal originating in the gut that primes the kidney for postprandial sodium excretion. Similar mechanisms may regulate the excretion of other dietary electrolytes, such as potassium [9].

The hormonal mediators of this gut–kidney interaction remain incompletely defined. Among gut hormones, glucagon-like peptide-1 (GLP-1) is of particular interest [9, 10]. GLP-1 is secreted by enteroendocrine L-cells in response to nutrient ingestion and exerts well-characterized metabolic effects, including glucose-dependent insulin secretion, suppression of glucagon, delayed gastric emptying, and central regulation of appetite and satiety [11]. In T2D, the incretin effect (i.e. the augmentation of insulin secretion by enteroendocrine hormones GLP-1 and GIP) is impaired, but can be restored by raising GLP-1 to therapeutical concentrations [12, 13].

GLP-1 is known to affect water and electrolyte balance [9, 14]. Emerging evidence suggests that dietary sodium intake may enhance GLP-1 secretion, which is thought to be mediated, at least in part, by sodium sensing through the sodium-glucose co-transporter-1 expressed on intestinal enteroendocrine cells [15]. Experimental and clinical studies showed that short-term treatment with GLP-1 receptor agonists (GLP-1 RA) increase fractional sodium excretion in the fasting and postprandial state in both healthy overweight volunteers and people with T2D. This may occur through inhibition of the proximal tubular sodium-hydrogen exchanger isoform-3 (NHE3), a transporter that is also expressed in the gut, and may regulate blood pressure [9, 14, 16, 17]. However, prior studies evaluating GLP-1RA effects on natriuresis did not standardize sodium intake or collect 24-h urine, limiting conclusions regarding overall sodium balances.

Given the impaired incretin response characteristic of T2D, we hypothesize that GLP-1-kidney feed-forward regulation of sodium excretion is attenuated in T2D, and that pharmacological GLP-1R activation would restore this response. We therefore conducted the REALITY trial to assess whether acute GLP-1RA exenatide infusion restores enhanced natriuresis following oral sodium loading compared to intravenous administration under standardized conditions.

## MATERIALS AND METHODS

### Trial design

The REALITY trial was a single-center mechanistic, randomized, double-blind, placebo-controlled three-period cross-over study designed to assess the effects of intravenous versus oral sodium loading, with or without concomitant GLP-1RA exenatide infusion, on cumulative 24-h sodium excretion and 24-h blood pressure in people with T2D. In addition, we included a cohort of healthy individuals who received an oral and intravenous sodium load, and urinary sodium excretion was quantified 2 h after administration.

The protocol was approved by the Medical Ethics Review Board of Amsterdam UMC (location VUMC, Amsterdam, The Netherlands), and registered at the International Clinical Trial Registry Platform (NL8293). The study was conducted in accordance with the Declaration of Helsinki and the International Conference on Harmonization of Good Clinical Practice. All participants provided written informed consent prior to any trial-related activity.

### Study population

Participants were recruited via advertisements in local newspapers, general practitioner offices, patient advocacy websites and the outpatient database of the Diabetes Centre Amsterdam. Eligible participants were men of European descent, aged 40–75 years, with established T2D [HbA1c: 42–86 mmol/mol (6.0–10.0%)] treated with stable doses of metformin with or without a sulfonylurea. A male cohort was chosen to limit variability in outcomes, as both renal sodium handling [18] and salt-sensitive blood pressure response [19] are known to be influenced by sex. In addition, growing evidence suggests that GLP1-RA response is also influenced by sex difference [20]. Inclusion required eGFR >60 ml/min/1.73 m^2^ and UACR ≤30 mg/mmol. Use of renin-angiotensin system inhibitors was permitted if the doses had been stable for ≥3 months. Key exclusion criteria were uncontrolled hypertension (office blood pressure >160/90 mmHg), recent (<3 months) major (cardiovascular) illnesses, significant chronic comorbidities, or use of medications known to affect sodium handling that could not be safely discontinued (including diuretics, thiazolidinediones, GLP-1RA, insulin, dipeptidyl peptidase-4 inhibitors, SGLT2-inhibitors, systemic glucocorticoids, immune suppressants, antimicrobial agents, chemotherapeutics, antipsychotics, tricyclic antidepressants, monoamine oxidase inhibitors). The healthy controls did not have chronic disease, did not use any medication and were to have normal glucose tolerance and kidney function.

### Intervention and randomization

Participants underwent three experimental conditions in random order: (1) intravenous sodium loading (ISL); (2) oral sodium loading (OSL) with placebo infusion (OSL-placebo), and (3) OSL with exenatide (OSL-exenatide). Treatment order was assigned using computer-generated block randomization with a 1:1:1 allocation ratio. The cross-over periods were separated by one-week intervals. On the OSL visits, participants received either exenatide (10 μg; dissolved in saline) or placebo (saline) administered intravenously in a total volume of 50 ml (equivalent to 7 mmol sodium) (46 ml of 0.9% NaCl + 4 ml autologous blood to prevent adsorption). The infusion was delivered at 15 ml/h for 30 min followed by 7.5 ml/h for 4.5 h. This regimen has previously been shown to achieve plasma exenatide concentrations within the therapeutic range (130–150 pg/ml) [21–23]. Study medication was prepared and administered by an independent physician to ensure blinding. OSL consisted of ingestion of a 300 ml sodium-rich, potassium-free herbal broth providing 150 mmol sodium, consumed within 30 min. During the ISL condition, an equivalent sodium load (300 ml 3% NaCl; 154 mmol sodium) was administered intravenously over 30 min.

The healthy controls received ISL and OSL in random order, administered in the same way as in the T2D participants.

### Trial procedures

To minimize variability in renal sodium handling, participants adhered to a sodium intake consistent with WHO-recommendations (<90 mmol sodium/day) for 7 days prior to the first experimental visit. They received consultations of the study dietician and physician. Compliance was verified by 24-h urinary sodium excretion, with 25% deviation from the indicated target being acceptable [24, 25]. Participants maintained this sodium intake throughout the 3-week study period. Participants were instructed to refrain from vigorous physical activity and alcohol for 24 h, and caffeine for 12 h before each study visit. On each test day, following an overnight fast, participants consumed 500 ml tap water to stimulate diuresis. All medication was postponed except metformin until completion of the study procedures. Upon arrival at the clinical trial unit (08:30 am), 24-h ambulatory blood pressure monitoring (ABPM) was initiated, with measurements obtained every 15-min during daytime and every 30 min overnight. Two venous cannulas were inserted: one in the dominant arm for iohexol infusion and study intervention administration, and one in the contralateral arm for serial blood sampling. After baseline blood collection, iohexol infusion was started concurrently with the sodium intervention. Iohexol (OmnipaqueTM, GE Healthcare B.V. Eindhoven, the Netherlands) was administered as a bolus of 36 mg/kg, followed by continuous infusion at 906 mg/h for 6 h to enable measurement of GFR. All procedures were conducted in a semi-recumbent position in a temperature-controlled room (22.0 ± 1.0°C). During the 6-h in-hospital phase, participants consumed 200 ml tap water hourly to maintain diuresis. Urine was collected via spontaneous voiding every 2 h, and venous blood samples were obtained hourly. Four hours into the in-hospital phase, participants consumed a sodium-free lunch provided by the study team. After completion of the in-hospital protocol, participants returned home to complete the 24-h urine collection. They were instructed to limit fluid intake to <1.5 l(water only), and to consume the standardized sodium-free dinner packages. Participants returned the following morning to submit urine collections and remove the ABPM device for read-out.

The healthy control group followed a similar preparation and sodium diet, but remained at the research unit for a follow-up time of 2 h. At this timepoint, urine was collected to assess urinary sodium excretion following ISL or OSL.

### Outcomes

The primary outcome was cumulative urinary sodium excretion after 2, 4, 6, and 24 h following each intervention. Secondary endpoints included 24-h blood pressure, fractional sodium excretion and measured GFR (mGFR). Exploratory endpoints comprised plasma electrolyte concentrations (measured hourly for 6 h), urinary electrolyte excretion at 2, 4, and 6 h, urinary volume, extracellular volume (ECV), osmolality parameters, and free water clearance (Fig. 1).

**Figure 1:**
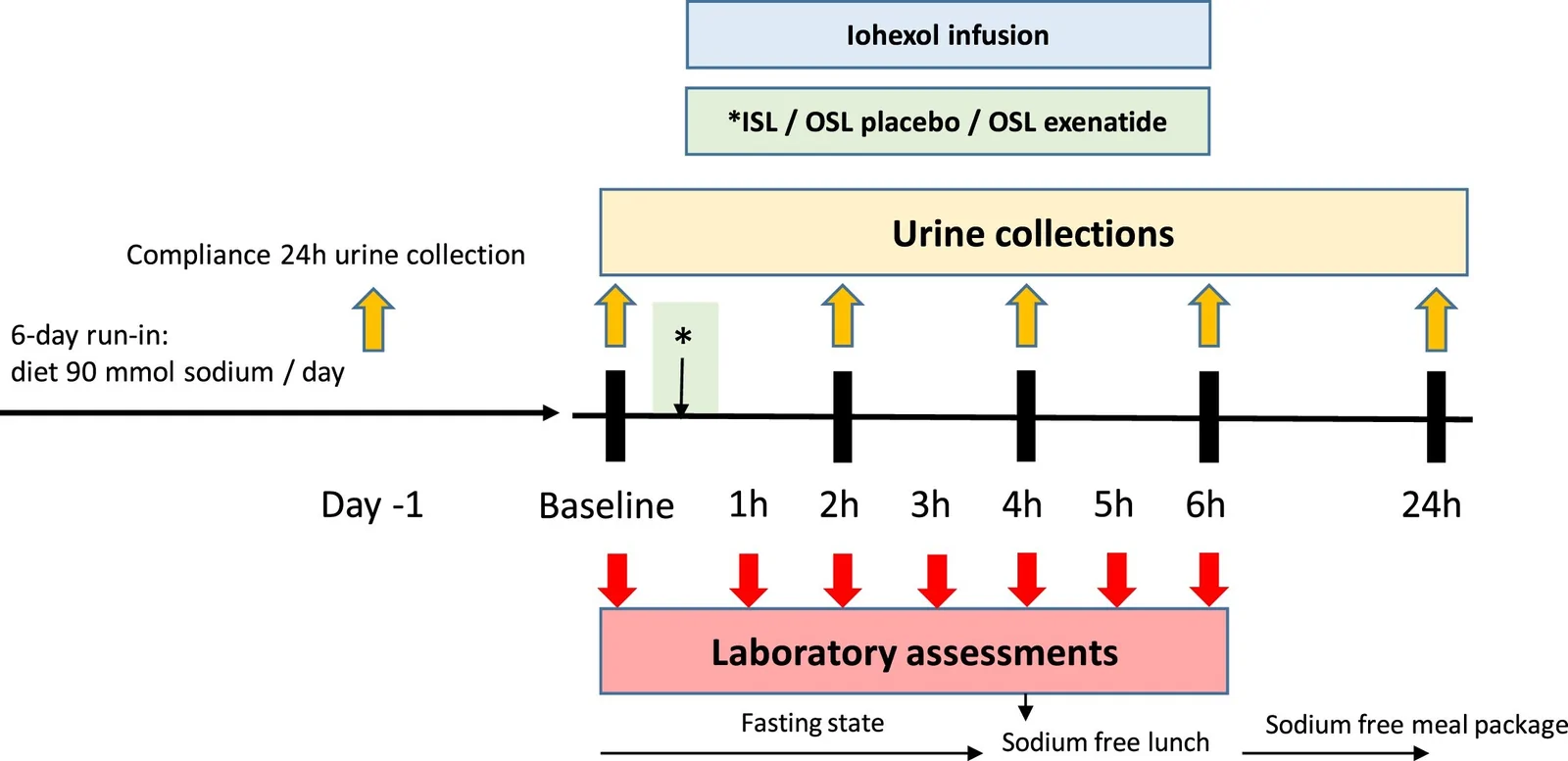
Design of the REALITY trial.

### Assays and calculations

Electrolyte measurements were performed by accredited clinical laboratories at Amsterdam UMC and Erasmus Medical Center Rotterdam. Venous plasma glucose concentrations were measured using a YSI-2300 STAT Glucose Analyzer (YSI Life Sciences, Yellow Springs, OH). Iohexol concentrations were quantified at the University Medical Center Groningen using liquid chromatography tandem mass spectrometry (TSQ Quantiva with UHPLC Vanquish, Thermo Fisher Scientific, Waltham, MA) [23]. eGFR was calculated by the CKD-EPI formula and mGFR was derived from timed-urine sampling as described [23]. Plasma sodium concentrations were determined by venous blood gas analysis and indirect ion selective electrode method. ECV was derived from iohexol distribution kinetics. Fractional sodium excretion was calculated using creatinine-adjusted urinary sodium concentrations. Urinary osmolality was derived from urinary sodium, potassium and urea; plasma osmolality from sodium, glucose and urea concentrations. Free water clearance was calculated as urine flow minus osmolar clearance [26].

### Statistical analysis

Sample size estimation was based on detecting a within-subjects difference of 15 mmol in cumulative 24-h sodium excretion between oral and ISL, assuming a standard deviation (±SD) 13 mmol, with 90% power and a two-sided alpha of 0.05. This calculation indicated that 11 participants were required. Statistical analyses were performed using SPSS version 28.0 (IBM Corporation, Chicago, IL). Continuous variables are presented as mean (±SD) for normally distributed data or median (IQR) for non-normal distributions. Normality was assessed by inspection of residual plots. Between-intervention comparisons were performed using two-way repeated measures analysis of variance (ANOVA). When main effects were statistically significant or showed a trend, pairwise comparisons of conditions at each time point were conducted with Bonferroni-adjusted post hoc tests. Non-normally distributed variables were log-transformed prior to analyses. A two-sided *P* value <.05 was considered statistically significant.

## RESULTS

### Characteristics of the study population

Between March 2021 and February 2022, 11 men with T2D completed the study. Baseline demographic and clinical characteristics are listed in Table 1. Participants were middle-aged (60 ± 5 years), overweight or obese (BMI 31.9 ± 3.9 kg/m^2^), and had had reasonably well-controlled glycemia on stable oral glucose-lowering therapy [HbA1c 7.5 ± 0.9% (58.9 ± 10.2 mmol/mol)]. Kidney function was preserved (eGFR 89 ± 5 ml/min/1.73 m²), and all participants had normoalbuminuria. Mean systolic blood pressure (SBP) and diastolic blood pressure (DBP) were 127 ± 22 mmHg and 18 ± 17 mmHg, respectively. Twenty-four-hour urinary sodium excretion before interventions averaged 82 ± 27 mmol/24-h, confirming adherence to the prescribed sodium-intake.

**Table 1:** Participant characteristics.

	Study participants (*N* = 11)
Age, yr	60 ± 5
Diabetes duration, yr	10.6 ± 6.3
Body weight, kg	104.1 ± 12.6
BMI, kg/m^2^	31.9 ± 3.9
Fasting plasma glucose, mmol/l	9.3 ± 1.3
HbA1c, %	7.5 ± 0.9
HbA1c, mmol/mol	58.9 ± 10.2
Hemoglobin, mmol/l	9.4 ± 0.5
Hematocrit, l/l	0.44 ± 0.02
eGFR (CKD-EPI), ml/min per 1.73 m^2^	89 ± 5
Participants with normoalbuminuria	11 (100%)
Systolic blood pressure, mmHg	127 ± 22
Diastolic blood pressure, mmHg	82 ± 17
Metformin use, *n* (%)	11 (100%)
Sulfonylurea use, *n* (%)	2 (18%)
Antihypertensive medication use, *n* (%)	7 (63.6%)
RASi use, *n* (%)	1 (9.1%)
24-h urinary sodium excretion, mmol	81.6 ± 26.6

Data are shown as number (%), mean with standard deviation (SD ±) or median with interquartile range. eGFR, estimated glomerular filtration rate; UACR, urine albumin-to-creatinine ratio. Of the patients using an anti-hypertensive agent, one used a blocker of the renin-angiotensin system.

### Effects of sodium loading on urinary electrolyte excretion

Cumulative sodium excretion over 24-h did not differ between ISL, OSL-placebo or OSL-exenatide (Fig. 2A, Table 2). Median cumulative 24-h sodium excretion was 104.5 (IQR 55.0–155.9) mmol during ISL, 100.5 (IQR 71.2–134.4) mmol during OSL-placebo, and 100.3 (IQR 63.2–123.1) mmol during OSL-exenatide (overall *P* > .05). Thus, 32%–35% of the sodium load was not excreted within 24 h, without differences between interventions. In contrast, in 10 healthy, lean controls (age 25 ± 5 years; eGFR 112 ± 9 ml/min/1.73 m^2^, HbA1c 5.3 ± 0.2%) who received equivalent sodium loads (154 mmol) in cross-over fashion, urinary sodium excretion was 100% higher following the OSL compared to the ISL 2 h after administration [6 (4–9) mmol vs. 12 (7–15) mmol, *P* = .042]. The additional urinary data from REALITY showed that urinary volume was not different after 2 h between the treatment arms. In addition, urinary volume over 24-h was similar across treatment conditions [ISL 3.1 l (2.6–3.6), OSL-placebo 2.7 L (2.1–3.2) and OSL-exenatide 2.5 l(2.2–3.0); *P* = .45] (Fig. 2B, Table 2). Baseline fractional sodium excretion was low and increased following sodium loading without between-group differences (Fig. 2C). Urinary electrolyte excretion at baseline and at 2, 4, 6 h did not differ between interventions (Table 2). Urinary pH levels were modestly higher during exenatide infusion, reaching statistical significance at 4 h compared with ISL (*P* = .03) (Table 2).

**Figure 2:**
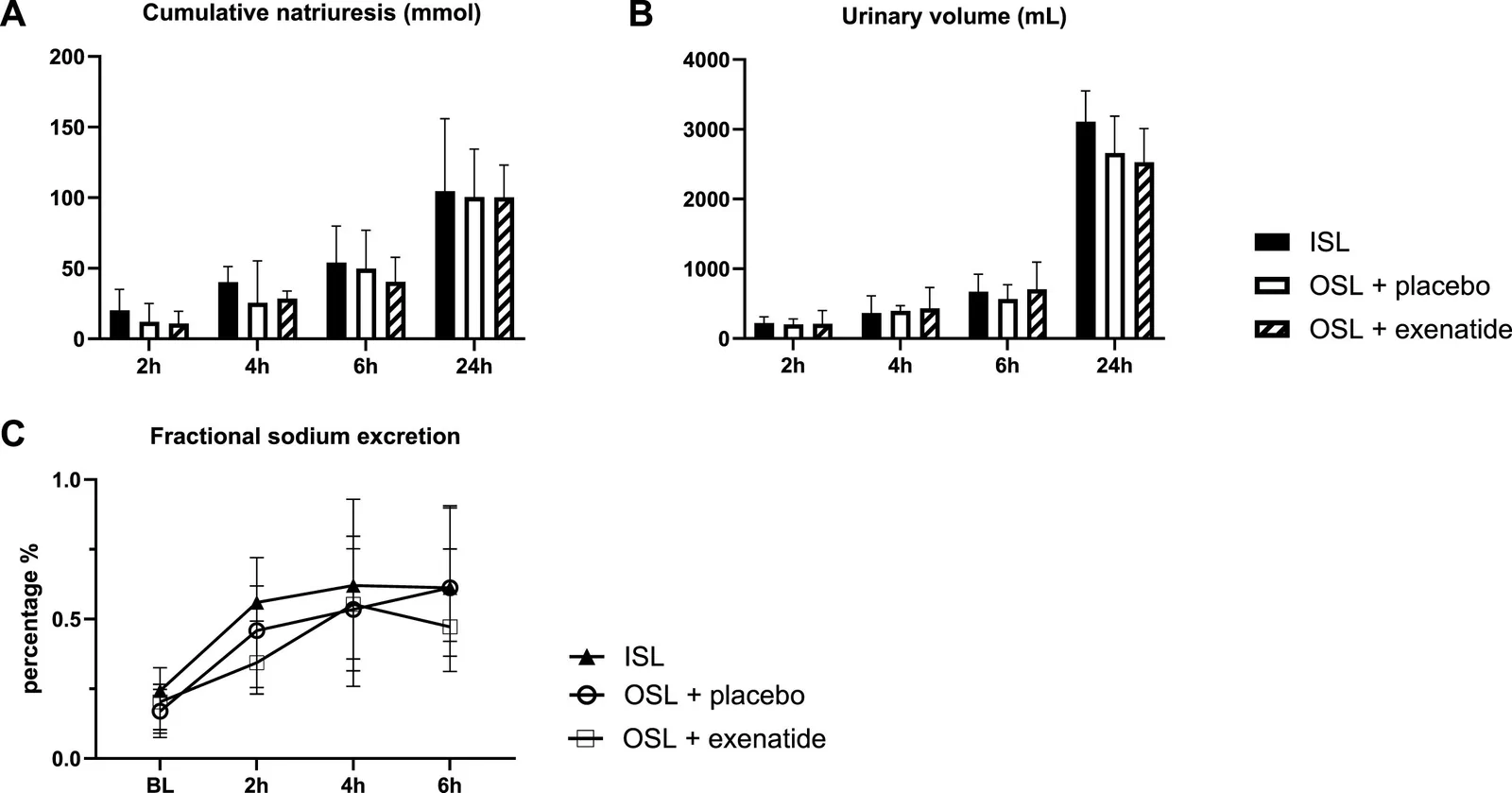
Effects of sodium loading on natriuresis, urine volume and fractional sodium excretion. Cumulative urinary sodium excretion (A) and cumulative urinary volume excretion (B) during 24 h after intravenous sodium load (black bars), oral sodium load with placebo (open bars) and oral sodium load with exenatide (hatched bars). Fractional sodium excretion (C) during 6 h after intravenous load (black triangle), oral sodium load with placebo (open circle) and oral sodium load with exenatide (open square). Data are shown as median with interquartile ranges due to unequal distribution of the data. Data were log-transformed to determine *P*-values with repeated measures ANOVA.

**Table 2:** Urinary electrolyte concentrations.

	Intravenous sodium load	Oral sodium load + placebo	Oral sodium load + exenatide	*P*-value pairwise comparison
Sodium (mmol), *P* = .65				
0–2 h	20.2 [7.6–35.0]	12.2 [8.3–25.1]	11.0 [7.7–19.5]	NA
0–4 h	40.2 [15.8–51.2]	25.7 [19.8–55.2]	28.6 [17.2–33.9]	NA
0–6 h	54.1 [26.8–80.0]	49.8 [31.9–77.0]	40.4 [29.1–57.8]	NA
0–24 h	104.5 [55.0–155.9]	100.5 [71.2–134.4]	100.3 [63.2–123.1]	NA
Potassium (mmol), *P* = .07				
0–2 h	13.6 ± 5.4	9.9 ± 3.6	11.6 ± 3.7	NA
0–4 h	26.6 ± 8.6	22.7 ± 6.4	25.4 ± 8.2	NA
0–6 h	36.1 ± 9.2	30.4 ± 7.9	34.5 ± 10.3	NA
0–24 h	67.9 ± 19.2	56.2 ± 9.9	55.8 ± 17.7	NA
Sodium/potassium ratio, *P* = .20				
0–2 h	1.6 ± 1.1	1.7 ± 0.9	1.3 ± 0.7	NA
0–4 h	1.4 ± 1.1	1.6 ± 1.2	1.6 ± 1.3	NA
0–6 h	1.9 ± 0.8	2.4 ± 0.8	2.0 ± 1.1	NA
0–24 h	1.5 ± 0.8	1.9 ± 0.8	1.8 ± 0.7	NA
Creatinine (mmol), *P* = .49				
0–2 h	1.8 ± 0.3	1.8 ± 0.5	1.8 ± 0.3	NA
0–4 h	3.3 ± 0.5	3.2 ± 0.6	3.3 ± 0.5	NA
0–6 h	4.8 ± 0.6	4.7 ± 0.8	4.9 ± 0.7	NA
0–24 h	12.9 ± 4.4	12.2 ± 3.5	12.5 ± 3.1	NA
Chloride (mmol), *P* = .24				
0–2 hrs	23.1 [10.4–40.8]	11.0 [9.8–24.8]	14.0 [10.9–19.4]	NA
0–4 h	45.3 [22.1–72.8]	30.3 [21.2–58.1]	28.0 [23.2–41.0]	NA
0–6 h	57.4 [34.6–99.3]	54.2 [34.1–79.9]	43.2 [34.7–57.9]	NA
0–24 h	113.7 [57.0–152.0]	99.0 [83.9–136.3]	85.8 [57.0–105.8]	NA
Urea (mmol), *P* = .57				
0–2 h	46.5 ± 8.5	45.1 ± 14.8	44.0 ± 13.2	NA
0–4 h	85.5 ± 13.7	83.9 ± 23.4	80.4 ± 22.2	NA
0–6 h	137.3 ± 20.5	128.6 ± 36.2	117.7 ± 31.5	NA
0–24 h	345.9 ± 126.8	344.9 ± 128.9	318.6 ± 100.3	NA
Urinary volume (ml), *P* = .55				
0–2 h	220 [135–310]	200 [150–280]	210 [150–400]	NA
0–4 h	365 [290–610]	395 [320–470]	430 [270–730]	NA
0–6 h	670 [520–920]	565 [500–770]	705 [375–1095]	NA
0–24 h	3108 [2573–3553]	2658 [2068–3188]	2528 [2178 -3013]	NA
Urine pH, *P* = .08				
Baseline	5.8 ± 0.7	5.8 ± 0.5	5.7 ± 0.5	NA
After 2 h	6.1 ± 0.7	6.0 ± 0.5	6.4 ± 0.7	NA
After 4 h	6.4 ± 0.6*	6.7 ± 0.6	7.2 ± 0.6*	0.03*
After 6 h	6.5 ± 0.8	6.7 ± 0.6	7.1 ± 0.7	NA

Data are in median with interquartile ranges or in mean with standard deviation (±). Unequally distributed data were log-transformed to determine *P*-values with repeated measures ANOVA (*P*-values reported on the row of the indicated variable). Where significance or a trend was observed, pairwise between condition comparisons were reported when significant; otherwise, results are shown as not applicable (NA); final column.

### Effects of sodium loading on plasma electrolyte concentrations

Plasma sodium concentrations significantly changed over time in all treatment arms (time effect *P* = .001). During OSL-exenatide, plasma sodium was transiently higher between 3 and 5 h compared with the other interventions (Fig. 3A). A similar pattern was observed for plasma chloride (Fig. 3B). Plasma potassium concentration was transiently higher during ISL in the first 3 h (Fig. 3C) [27]. Exenatide infusion lowered blood glucose concentrations compared with both other conditions (Fig. 3D). No significant differences were observed in other measured electrolytes (Fig. 3E–G).

**Figure 3:**
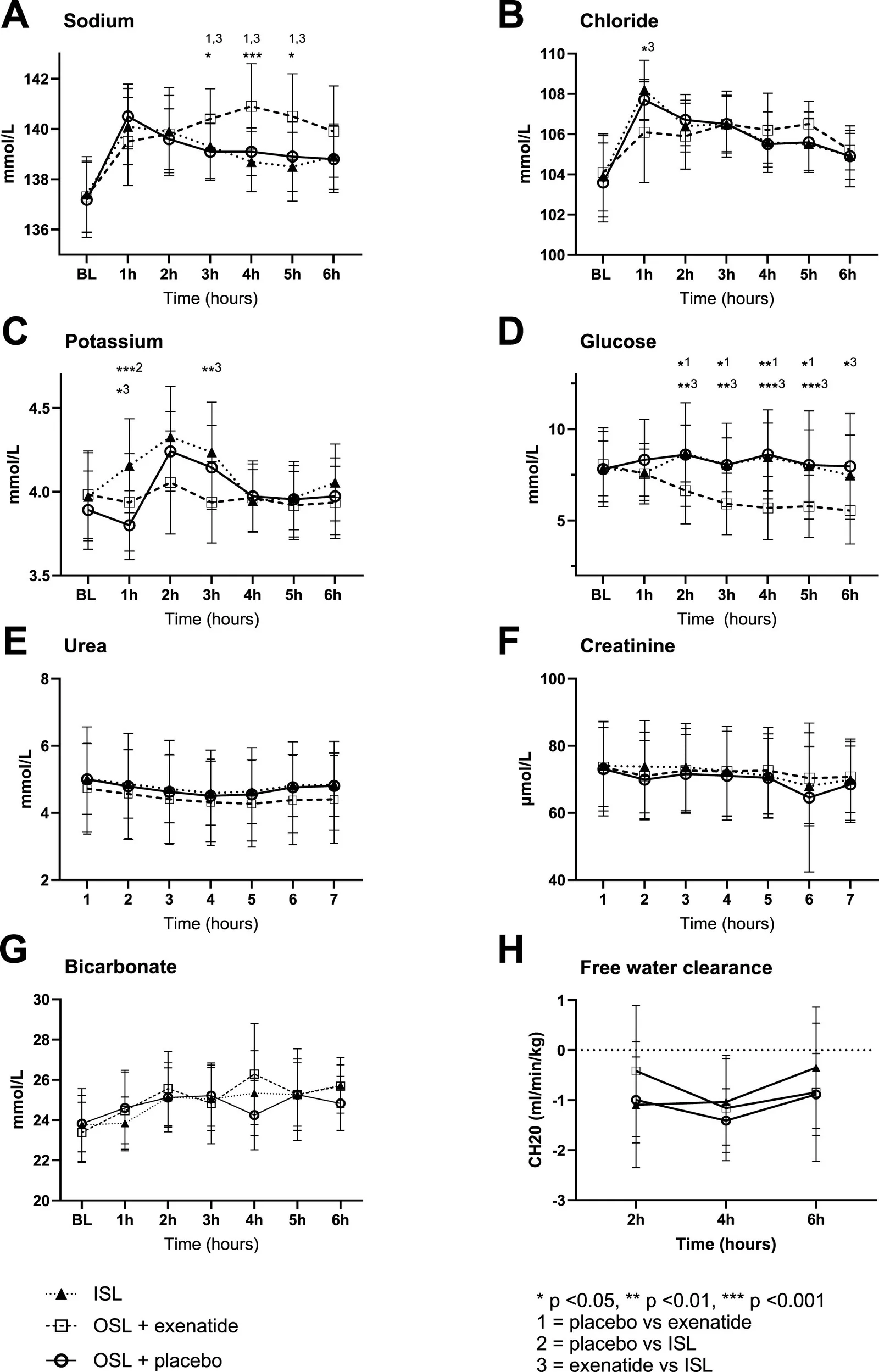
Effects of sodium loading on plasma electrolytes and free water clearance. (A–G) Serum electrolyte concentrations (mean ± SD) from baseline to 6 h after interventions. (H) Free water clearance (ml/min/kg) from 2 to 6 h. Intravenous load (black triangle), oral sodium load with placebo (open circle) and oral sodium load with exenatide (open square).

### Effects of sodium loading on free water clearance

Because plasma sodium trajectories differed during exenatide infusion, free water clearance was evaluated. At 2 h, free water clearance tended to be higher during OSL-exenatide (−0.4 ± 1.3 ml/min/kg) compared with OSL-placebo (−1.0 ± 0.9 ml/min/kg) and ISL (−1.1 ± 1.3 ml/min/kg) although this did not reach statistical significance (Fig. 3H).

### Effect of sodium loading on systemic and kidney hemodynamic function

Acute exenatide administration did not change systemic hemodynamic function. Daytime 24-h ambulatory SBP was 132 ± 14 during ISL, 128 ± 16 during OSL-placebo and 130 ± 11.8 mmHg during OSL-exenatide. Nighttime SBP were similarly comparable across conditions. No differences were observed in DBP, nocturnal dipping or heart rate (Table 3). Each sodium loading intervention was associated with a small, not significant, increase in mGFR, without differences between groups. Similarly, ECV remained unchanged across all study conditions (Table 3).

**Table 3:** Blood pressure, mGFR and extracellular volume responses.

	Intravenous sodium load	Oral sodium load with placebo	Oral sodium load with exenatide	*P*-value
24 h ABPM				
- Systolic BP, mmHg Daytime Nighttime	132 ± 14 113 ± 13	128 ± 16 111 ± 16	130 ± 12 115 ± 13	0.31 0.25
- Diastolic BP, mmHg Daytime Nighttime	87 ± 9 72 ± 9	85 ± 10 70 ± 11	87 ± 8 73 ± 10	0.33 0.19
- Dipping, % Systolic BP	13 ± 4	13 ± 4	12 ± 6	0.56
- Heartrate, BPM Daytime Nighttime	76 ± 12 68 ± 9	77 ± 10 67 ± 9	78 ± 10 68 ± 8	0.48 0.92
ECV (l)	29.2 ± 9.9	30.7 ± 11.8	27.6 ± 11.6	0.25
mGFR (ml/min)				
2 h–4 h	115.2 ± 24.7	120.2 ± 18.1	113.4 ± 18.5	0.27
4 h–6 h	129.9 ± 20.7	121.8 ± 20.9	121.4 ± 19.6	0.16

Data are in mean with standard deviation (±). ABPM, ambulatory blood pressure measurement; ECV, extracellular volume; mGFR, measured glomerular filtration rate.

## DISCUSSION

The gastrointestinal tract contributes to a feedforward signal promoting postprandial urinary sodium excretion, as evidenced by more rapid urinary sodium clearance following oral versus intravenous sodium loads [6–8]. GLP-1 has been proposed as a candidate mediator of this gut-kidney axis. In the present study, we provide the first evidence that this feed-forward mechanism is impaired in individuals with T2D, studied under standardized sodium conditions. Importantly, acute infusion of the GLP-1RA exenatide failed to restore this gut-kidney interaction, in contrast to its established ability to enhance the defective incretin effect in this condition. Together, these findings suggest that feed-forward gut-kidney signaling of sodium handling is impaired in T2D, and that acute GLP-1R-stimulation alone is insufficient to normalize this pathway.

Human physiology experiments dating back several decades showed that oral sodium ingestion can produce a more rapid natriuretic response than an equivalent intravenous load, supporting the existence of a gastrointestinal feed-forward signal to the kidney [6–8]. These studies demonstrated that urinary sodium excretion was higher after OSL versus ISL, which we observed in our healthy controls as well. This phenomenon has been interpreted as evidence for gut or portal sodium sensing that primes renal excretion independent of simple volume expansion. In contrast, our participants with T2D showed no such divergence, indicating loss or attenuation of this feed-forward mechanism. T2D is characterized by altered renal sodium handling, insulin resistance, and autonomic dysregulation—all factors that may blunt coordinated gut-kidney communication. Our findings therefore support the hypothesis that metabolic disease disrupts integrated sodium regulatory pathways that are preserved in health.

In this regard, GLP-1 has been proposed as a candidate mediator of gut-kidney communication because of its natriuretic properties and its established role in postprandial physiology. Experimental and clinical studies indicate that GLP-1R-activation can inhibit proximal tubular sodium reabsorption, at least in part via modulation of the NHE3, and may transiently increase GFR [9]. Together, these actions raise the possibility that GLP-1 contributes to the physiological signal linking nutrient ingestion to renal excretion. Despite this rationale, and in contrast to our formulated hypothesis, acute exenatide infusion did not restore this apparent impairment of the gut-kidney axis in T2D. This suggests that gut-kidney signaling likely involves broader enteroendocrine networks beyond GLP-1 alone. Accordingly, other gut-derived hormones, including gastrin, ghrelin, vasoactive intestinal polypeptide, guanylin, uroguanylin, peptide YY, amylin or secretin, have been implicated in sodium balance and may act synergistically with neural reflex pathways in physiological gut-kidney signaling [9, 28].

In addition, the composition of nutrients reaching the gut influence the feed-forward gut-kidney response, such that renal sodium handling depends not only on sodium content but also on overall dietary context [28]. Further clarification of meal-induced enteroendocrine hormone release is therefore important, as impaired sodium sensing may contribute to meal-related salt-sensitive hypertension in T2D, potentially promoting both cardiovascular and kidney disease [9]. Our findings contrast with several reports suggesting that GLP-1RA promote natriuresis, which contribute to their cardiorenal protective profile, including blood pressure-lowering [29–32]. While some short-term studies have demonstrated transient increases in sodium excretion [23, 33–37], longer-term studies generally show no sustained natriuretic effect [14, 38]. The mechanisms underlying GLP-1RA-induced natriuresis remain uncertain, particularly since GLP-1 receptors are not clearly expressed within the tubular cells. However, in rodent studies, GLP-1RA treatment was shown to inhibit NHE3 activity in the proximal tubule, thereby reducing proximal sodium reabsorption and hydrogen secretion [39]—a mechanism we previously confirmed in humans [14]. Supporting this concept, GLP-1R-activation is consistently associated with increased urinary pH in both previous [23, 35, 36, 40] and in current study. Importantly, compensatory adaptations likely limit sustained sodium loss during chronic GLP-1R-activation, with distal nephron segments increasing sodium reabsorption to preserve balance. Similar homeostatic responses are observed with other natriuretic drugs, including SGLT-2 inhibitors [41] and loop diuretics [42] where distal tubular compensation attenuates long-term natriuresis. Another proposed mechanism by which GLP-1 may acutely facilitate postprandial electrolyte clearance is through transient increases in GFR driven by nitric-oxide mediated effects on the endothelium [34]. However we observed no kidney hemodynamic effect in participants with T2D, consistent with our previous study in this population [23]. Notably, many studies reporting acute natriuretic effects of GLP-1RAs did not standardize sodium intake, making it difficult to distinguish pharmacological effects from variations in dietary sodium exposure—a limitation addressed in the present protocol.

We assessed the effects of the interventions on 24-h blood pressures responses. Exenatide did not alter SBP, DBP, heart rate or ECV. These findings contrasts with longer-term studies in which GLP-1RAs lower blood pressure, effects that cannot be fully attributed to weight loss alone [29, 30]. The mechanisms underlying these chronic blood pressure benefits remain incompletely understood. The fact that GLP-1R-activation was also shown to acutely increase blood pressure, possibly mediated by sympathetic nervous system activation, could have complicated the interpretation of our results [9, 23, 37].

Administration of oral versus ISL did not differentially influence urinary excretion of other electrolytes or urine volume, consistent with previous observations in healthy individuals [6, 7]. Exenatide tended to increase urinary volume excretion during the first 6 h, although between-group differences were not statistically significant. Plasma sodium concentrations were modestly higher during exenatide exposure. Aside from its expected glucose-lowering effect (confirming pharmacological activity), exenatide did not alter other plasma electrolytes.

Several limitations should be considered when interpreting these findings. First, our study was designed as a mechanistic physiological investigation with a modest sample size, which limits statistical power for secondary and exploratory outcomes and may reduce sensitivity to detect subtle treatment effects. Second, the study population consisted exclusively of men with preserved kidney function and well-controlled T2D treated with metformin and sulfonylureas, enhancing internal consistency but limiting generalizability to broader patient populations, including women, individuals with advanced chronic kidney disease or those using insulin or SGLT2-inhibitors. Third, the intervention involved short-term infusion of exenatide, a first-generation GLP-1RA selected for its acute mode of action. Longer-acting, modern GLP-1RAs, such as once-weekly semaglutide, or chronic drug-exposure, may produce adaptive physiological responses, that differ from those observed in this acute setting. Fourth, we did not measure fecal sodium excretion, which could be relevant due to the intestinal NHE3 transporter that is potentially affected by GLP-1RA treatment. We also did not quantify sodium sequestration in tissues such as skin or the glycocalyx. Finally, our healthy control comparison group consisted of younger individuals and included women, in contrast to our male T2D cohort, which limits comparability between groups.

In conclusion, feed-forward sodium sensing from gastrointestinal tract to the kidneys appears to be impaired in people with T2D, paralleling the well-described defect in incretin signaling. Acute GLP-1R-activation with exenatide did not restore this gut-kidney natriuretic response, suggesting that this physiological pathway likely depends on broader enteroendocrine and neural networks rather than GLP-1 signaling alone. Future compounds combining GIP and glucagon receptor agonism might have differential effects on sodium balance. The specific contribution of GIP receptor agonism to natriuresis remains unclear thus far, whereas glucagon has been shown to induce a mild natriuretic effect in rodents [43]; however, clinical research in humans is still lacking. Thus, clinical research regarding these dual and triple therapies in relation to the endpoints presented here is warranted. Clarifying the mechanisms governing gut-kidney communication could redefine our understanding of sodium regulation in metabolic disease and support the development of targeted therapies designed to restore physiological sodium sensing, with potential benefits for salt-sensitive hypertension and cardiorenal protection.

## Data Availability

The data that support the findings of this study are available from the corresponding author upon reasonable request.
